# The Multifaceted Roles of the BCL-2 Family Member BOK

**DOI:** 10.3389/fcell.2020.574338

**Published:** 2020-09-15

**Authors:** Samara Naim, Thomas Kaufmann

**Affiliations:** Institute of Pharmacology, University of Bern, Bern, Switzerland

**Keywords:** apoptosis, BCL-2 family, Bok, cell death, cancer, metabolism, autophagy, er stress

## Abstract

BCL-2–related ovarian killer (BOK) is—despite its identification over 20 years ago—an incompletely understood member of the BCL-2 family. BCL-2 family proteins are best known for their critical role in the regulation of mitochondrial outer membrane permeabilization during the intrinsic apoptotic pathway. Based on sequence and structural similarities to BAX and BAK, BOK is grouped with these “killers” within the effector subgroup of the family. However, the mechanism of how exactly BOK exerts apoptosis is not clear and controversially discussed. Furthermore, and in accordance with reports on several other BCL-2 family members, BOK seems to be involved in the regulation of a variety of other, “apoptosis-independent” cellular functions, including the unfolded protein response, cellular proliferation, metabolism, and autophagy. Of note, compared with other proapoptotic BCL-2 family members, BOK levels are often reduced in cancer by various means, and there is increasing evidence for BOK modulating tumorigenesis. In this review, we summarize and discuss apoptotic- and non–apoptotic-related functions of BOK, its regulation as well as its physiological and pathophysiological roles.

## Introduction: The BCL-2 Protein Family in Apoptosis

Apoptosis, the first described and probably best understood form of programed cell death, is a physiological process that actively leads to the death of a cell. Apoptosis is critical for the removal of old, unwanted or critically damaged cells, both during development and for the maintenance of cellular homeostasis in adult multicellular organisms. Its regulation reaches the complexity of cell growth and cell proliferation, and a fine-tuned and self-regulatory balance between cell growth and cell death guarantees the healthy state of individual organisms (Singh et al., 2019). Cellular turnover in adult humans is estimated to be around 1 million per second (Reed, 2006). Given the massive number of cells constantly dying in our bodies, it is not surprising that nature has ensured a highly efficient, fast, and safe removal process of dying cells. However, there is a strong connection between the imbalance in apoptosis regulation and various pathophysiologies (Favaloro et al., 2012). Many cancer cells, for example, increase their prosurvival activity during the process of malignant transformation or in the course of radiotherapeutic/chemotherapeutic intervention, contributing to the development of resistance against apoptotic stimuli. One commonly observed way to achieve this is through upregulation of antiapoptotic BCL-2 family members, such as BCL-2, BCL-XL, or MCL-1 (Campbell and Tait, 2018). To overcome such apoptosis resistance of cancer cells, great interest in developing specific inhibitors of antiapoptotic BCL-2 proteins, so-called BH3 mimetics, has arisen over the past years, with a first compound [the BCL-2–specific inhibitor venetoclax (Venclexta)] approved by the US Food and Drug and Administration for chronic lymphocytic leukemia and acute myeloid leukemia (Montero and Letai, 2018; Timucin et al., 2019). On the other hand, excessive apoptosis is described in neurodegenerative diseases such as Alzheimer and Huntington disease (Mattson, 2000; Ghavami et al., 2014).

Central to apoptosis induction are cysteine aspartate–specific proteases, called caspases. Caspases form an evolutionary conserved family of cysteine proteases involved in cell death (apoptotic caspases), as well as inflammatory responses (inflammatory caspases) (Zheng et al., 1999; Van Opdenbosch and Lamkanfi, 2019). Apoptotic caspases can be subdivided into initiator (caspases-8, -9, and -10) and effector caspases (caspases-3, -6, and -7) (Van Opdenbosch and Lamkanfi, 2019). Caspases are mainly abundant in their inactive state as procaspases (Mcilwain et al., 2013). Once autoactivated following intrinsic or extrinsic initiation of apoptosis, initiator caspases activate effector caspases through proteolytic cleavage (Shi, 2004). Activated effector caspases then cleave cellular substrates leading to cell death (Julien and Wells, 2017).

Members of the BCL-2 family are central regulators of the so-called intrinsic, or mitochondrial, apoptotic pathway, a pathway that can be activated in possibly all cell types in response to a plethora of intrinsic stress (“apoptotic”) stimuli. These include DNA damaging insults, oxidative stress, aberrant calcium fluxes, endoplasmic reticulum (ER) stress, nutrient deprivation, or infection by pathogens, among others (Lopez and Tait, 2015). Once activated, the intrinsic apoptotic pathway results in the BCL-2 family regulated discrete permeabilization of the mitochondrial outer membrane (MOMP), with subsequent release of many proteins from the mitochondrial intermembrane space into the cytoplasm. Among the latter are apoptogenic proteins (such as cytochrome c or SMAC/DIABLO), which are needed for—or facilitate—the downstream activation of apoptotic caspases (Lopez and Tait, 2015).

The members of the BCL-2 family are small globular proteins that contain up to four rather loosely conserved Bcl-2 homology (BH) domains, BH1–BH4. All family members contain the hydrophobic BH3 domain, which is important for protein–protein interaction between the various BCL-2 proteins, and most members contain a C-terminal α-helical transmembrane domain (TMD) that serves to tail-anchor the protein to intracellular membranes (Schinzel et al., 2004; Wilfling et al., 2012). Based on structural and functional homologies, the family is classified into three subgroups: the antiapoptotic proteins (BCL-2, BCL-XL, BCL-W, MCL-1, BFL-1/BCL-2A1, and BCL-B), the proapoptotic BH3-only proteins (BAD, BID, BIK, BIM, BMF, HRK, NOXA, PUMA), and the proapoptotic effector proteins BAX, BAK, and BCL-2–related ovarian killer (BOK) (Adams and Cory, 2018).

The interaction of the BCL-2 proteins depends on the abundance, the attraction, and the affinity of the proteins. The affinity between different proteins on its part results from conformational changes of the proteins (Kale et al., 2018). There are three main types of interaction: (1) binding of the BH3 domain of so-called “activator” BH3-only proteins (such as BIM or BID) to the BH3-binding groove of the effectors BAX and/or BAK, leading to their direct activation; (2) binding of the BH3-binding groove of antiapoptotic proteins to the BH3 domain of BAX/BAK or activator BH3-only proteins, leading to their sequestration; (3) binding of the BH3 domain of so-called “sensitizer” BH3-only proteins (such as BAD or NOXA) to the BH3-binding groove of antiapoptotic proteins, leading to their inactivation (Kale et al., 2018).

The ultimate goal of the early initiation phase of intrinsic apoptosis is MOMP. Whether or not MOMP occurs depends on the ratio between proapoptotic and antiapoptotic proteins, or more precisely, on their activation status and the interactions between them (Kale et al., 2018). Equally important seems the composition of the lipid bilayer of the mitochondrial outer membrane (MOM), where most of the previously described interactions take place and which has an active role in facilitating structural changes of the BCL-2 proteins, resulting in affinity changes and consequently alterations of interactions (Kale et al., 2018).

Once activated, the effector proteins BAX and BAK oligomerize to form pores leading to MOMP (Cosentino and Garcia-Saez, 2017), a process described as point of no return during the initiation of intrinsic apoptosis. While BAK is already located at the MOM, BAX translocates from the cytosol to the mitochondrial membrane upon activation (Wolter et al., 1997; Todt et al., 2015).

Whereas regulation of the intrinsic apoptotic pathway, specifically MOMP, is the best understood role of BCL-2 family members, it is important to mention that for most—if not all—members, other, distinct non-apoptotic roles have been described. These range from modulating mitochondrial shape and metabolism, calcium flux, the unfolded protein response (UPR), DNA damage response, glucose and lipid metabolism to cellular proliferation and autophagy [reviewed in (Gross and Katz, 2017)].

## A Short History of BOK

BOK was first discovered in 1997 by Hsu et al. (1997) in a yeast two-hybrid screen of a rat ovarian fusion library using MCL-1 as a bait and by Inohara et al. (1998) by sequence homology prediction screening, naming it “matador” (MTD), meaning *killer* in Spanish. Hsu et al. (1997) initially described BOK as highly and restrictedly expressed in rat ovaries, testis and uterus, hence the name “Bcl-2–related ovarian killer.” Within the first years after its discovery, only a few articles on BOK were published. Besides classifying it as a proapoptotic BCL-2 protein based on transient overexpression experiments and describing first interaction partners (MCL-1, BHRF1, and BFL-1, but not BCL-2, BCL-XL, or BCL-W) (Hsu et al., 1997), sequence analyses revealed BOK as an evolutionarily highly conserved BCL-2 member (Zhang et al., 2000). Not only was it reported to be important for apoptosis regulation in normal B-cell development in chicken (Brown et al., 2004) but also for the increased trophoblast cell death found in preeclampsia, regulated by the MCL-1/MTD (BOK) rheostat (Soleymanlou et al., 2005; Soleymanlou et al., 2007). The interest in BOK rapidly increased in 2010 upon the discovery that the locus containing the *BOK* gene is frequently deleted across human cancers, thus indicating a possible tumor suppressor role for BOK (Beroukhim et al., 2010) as well as the characterization of the first *Bok*-deficient mouse model in 2012 (Ke et al., 2012). This latter study showed that the expression of BOK is, in contrast to earlier assumptions (Hsu et al., 1997), not restricted to reproductive organs but rather widely distributed across tissues, in which it is detectable both at mRNA and protein levels (Ke et al., 2012). Because of its proapoptotic potential and its considerable amino acid sequence homologies with BAX and BAK, BOK was clustered in the effector (“killer”) subgroup of the BCL-2 family of proteins (Hsu et al., 1997). Until recently, there was no structural information available on BOK. The structural similarities between BOK and BAX/BAK were substantiated in 2018 by two independent studies using X-ray crystallography on chicken BOK and NMR spectroscopy on human BOK, respectively (Ke et al., 2018; Moldoveanu and Zheng, 2018; Zheng et al., 2018). However, as discussed in more detail below, there are several important differences that distinguish BOK properties and functions from those of BAX or BAK. More than 20 years after its discovery, the role of BOK in apoptosis is not entirely understood and to some extent controversially discussed (Yakovlev et al., 2004; Jaaskelainen et al., 2010; Carpio et al., 2015; D’Orsi et al., 2016; Einsele-Scholz et al., 2016; Llambi et al., 2016; Fernandez-Marrero et al., 2017; Moldoveanu and Zheng, 2018; Schulman et al., 2019). There is increasing evidence supporting roles of BOK in cellular functions other than cell death regulation, namely, uridine metabolism and cellular proliferation (Ray et al., 2010; Moravcikova et al., 2017; Rabachini et al., 2018; Srivastava et al., 2019), autophagy (Kalkat et al., 2013), mitochondrial physiology (D’Orsi et al., 2017; Ausman et al., 2018; Schulman et al., 2019), ER homeostasis, and modulation of the UPR (Echeverry et al., 2013; Carpio et al., 2015; Schulman et al., 2016; Moravcikova et al., 2017; Kang et al., 2019). Thus, there are many open questions remaining to fully understand the different roles and functions of this protein.

## BOK Gene Targeting in Mice

The first *Bok^–/–^* mouse was published in 2012 by Ke et al., using homologous recombination in C57BL/6-derived embryonic stem cells targeting half of exon 1 and exon 2 of *Bok* (with exon 2 containing the ATG start site) (Ke et al., 2012). BOK knockout mice did not show an overt phenotype and were produced at the expected Mendelian frequency (Table 1; Ke et al., 2012). Investigated organs of *Bok^–/–^* mice appeared normal, and there was no difference in hematopoietic cell subset composition (Ke et al., 2012). An important message of this study was that BOK is widely expressed in mouse tissues and readily detectable at the protein level in most tissues analyzed. Expression in the bone marrow, however, was shown to be low. Among the hematopoietic subsets, BOK expression was highest in myeloid cells but very low in lymphoid cells. These expression profiles may explain the finding that loss of BOK did not accelerate lymphoma development in Eμ-myc transgenic mice (Ke et al., 2012). Along the same line, *Bok*-deficient lymphoid and myeloid cells were found to respond normally to the classic apoptotic stimuli etoposide, dexamethasone, FasL, or the BH3 mimetic ABT-737 (Ke et al., 2012).

**TABLE 1:** *Bok*-deficient mouse models.

**Model**	**Targeting strategy/comments**	**Characteristics**	**References**
*Bok****^–/–^***	• Homologous recombination in C57BL/6 derived ES cells; C57BL/6J genetic background • Deletion of half of exon 1 and exon 2	• No overt phenotype • Reproduction at expected Mendelian frequency	Ke et al., 2012
*Bok****^–/–^***	• Homologous recombination in 129-derived ES cells; backcrossed to C57BL/6 genetic background • Deletion of exons 2 and 3	• Normal development	Carpio et al., 2015
*Bok****^–/–^***	• Homologous recombination in 129/SvEv derived ES cells; backcrossed to C57BL/6N genetic background • Deletion of exons 2–5	• No overt phenotype	Llambi et al., 2016
*E*μ*-myc/Bok****^–/–^***	• Crossing *E***μ***-myc* transgenic mice with full-body *Bok^–/–^* mice (Ke et al., 2012)	• No acceleration of lymphoma development and no impact on disease severity compared to *E***μ***-myc* transgenic mice	Ke et al., 2012
Nup98-HoxD13 (NHD13)/*Bok****^–/–^***	• Crossing NHD13 transgenic mice with full-body *Bok^–/–^* mice (Carpio et al., 2015)	• Acute myeloid leukemia development in 36.7% of mice • Development of progressive anemia: lower hemoglobin, lower mean cell hemoglobin concentration, higher mean cell volume	Kang et al., 2019
*Bok^–/–^Bak^–/–^ DKO*	• Crossing *Bak^–/–^* mice with *Bok^–/–^* mice	• No noticeable defects compared to *BAK^–/–^* single knockout	Ke et al., 2013
*Bok^–/–^Bax^–/–^ DKO*	• Crossing *Bax^–/–^* mice with *Bok^–/–^* mice	• Increased ovarian follicle numbers at 1 year of age	Ke et al., 2013
*Bok^–/–^Bax^–/–^Bak^–/–^ TKO* in the hematopoietic system	• Bone marrow chimeras obtained by injection of TKO fetal liver cells (FLC, E14) into lethally irradiated wild-type recipients	• Slightly more severe phenotype compared to mice reconstituted with FLCs of *Bax^–/–^Bak^–/–^ DKO* mice • Increased numbers of leukocytes in the periphery, as well as in several organs • Lymphocytic infiltration in multiple organs and organ-specific autoantibodies	Ke et al., 2015
*Bok^–/–^Bax^–/–^Bak^–/–^* full-body *TKO*	• Crossing *Bok^–/–^* (Ke et al., 2012), *Bax^–/–^* (Knudson et al., 1995) and *Bak^–/–^* (Lindsten et al., 2000) mice; C57BL/6J genetic background	• 1% of mice survive into adulthood • Developmental defects at E11.5 • Multiple midline defects at E18.5 • Pups develop abnormal tissue growth in multiple locations	Ke et al., 2018

Lack of a spontaneous phenotype was confirmed in two independently derived *Bok*-deficient mouse models (Carpio et al., 2015; Llambi et al., 2016). This finding was no great surprise, also given that neither BAX nor BAK single knockout mice display major phenotypes, with the exception of testicular atrophy and infertility seen in *Bax^–/–^* males (Knudson et al., 1995; Lindsten et al., 2000).

While loss of either BAK or BAX alone is compatible with the development of mice, *Bax^–/–^Bak^–/–^* double-knockout (DKO) mice are obtained at less than 10% of the expected Mendelian frequency, and only few survive into adulthood (Lindsten et al., 2000; Mason et al., 2013). Those surviving mice develop severe neurological and hematopoietic abnormalities, splenomegaly, and lymphadenopathy (Lindsten et al., 2000). On the other hand, combined gene knockouts of *Bok* and *Bax*, or *Bok* and *Bak*, respectively, did not worsen the phenotype of *Bak^–/–^* or *Bax^–/–^* single knockout mice, except for an increased oocyte count in *Bax^–/–^Bok^–/–^* DKO females (Ke et al., 2013). Mice lacking BAX, BAK, and BOK [triple-knockout (TKO)] in the hematopoietic system showed a slightly more severe phenotype compared to respective *Bax^–/–^Bak^–/–^* DKO controls, including increased levels of autoantibodies and leukocyte infiltration in several organs (Ke et al., 2015). These data were an early indication that BOK may have redundant functions with BAX and BAK. The proof that BOK indeed has overlapping functions with BAX and BAK also beyond the hematopoietic compartment came with the generation of full-body *Bax^–/–^Bak^–/–^Bok^–/–^* TKO mice in 2018 by Ke et al. (2018). This article showed that TKO mice develop more severe defects and die earlier compared with *Bax^–/–^Bak^–/–^* DKO mice, with 99% developing lethal abnormalities. With developmental defects starting from E11.5, TKO pups at E18.5 showed, among others, multiple midline defects and aortic arch defects, as well as abnormal tissue growth in multiple organs, all of which were thought to be a consequence of inactivated intrinsic apoptosis (Ke et al., 2018). Besides providing the current best evidence for a physiologically relevant role of BOK in apoptosis and embryogenesis, this study also demonstrated that multiple organs develop more or less normally in TKO mice and that a small proportion (1%) of TKO mice even survives into adulthood, indicating that intrinsic apoptosis is not strictly required for the development of mice (Ke et al., 2018).

## Interaction Partners and Subcellular Localization of BOK

**FIGURE 1:**
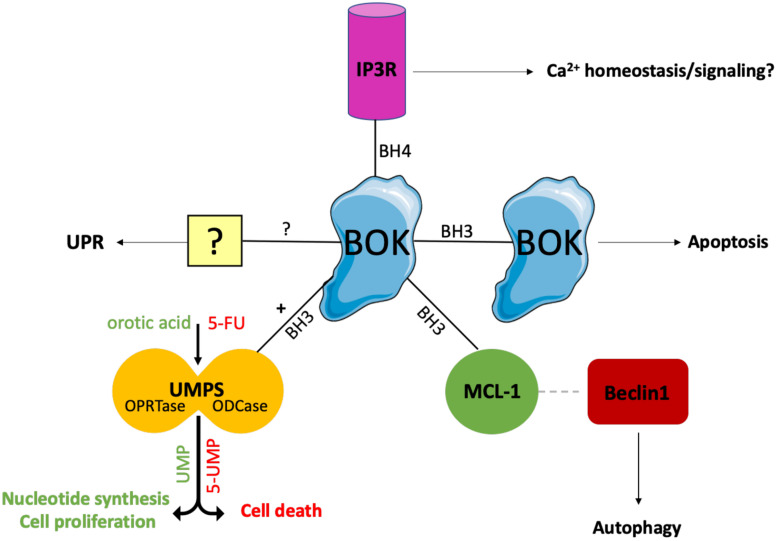
Interaction partners of BOK. Only few BOK interacting partners have been described so far. Using yeast two-hybrid screens, BOK was found to interact with MCL-1. This interaction has been proposed to promote autophagy, as BOK binding to MCL-1 frees MCL-1–bound Beclin-1, which in turn initiates the autophagy machinery. Moreover, BOK is constitutively bound to IP3Rs (IP3R1 and IP3R2) at the ER via its BH4 domain. IP3Rs may act as a regulatory sink for BOK to keep the levels of unbound (“free”) BOK low. Furthermore, co-immunoprecipitation experiments indicated that BOK may interact with itself in a BH3 domain–dependent manner. Whether BOK truly forms dimers or homo-oligomers and whether this oligomerization induces MOMP need to be further investigated. Recently, BOK was found to interact with and increase the activity of the enzyme UMPS via its BH3 domain. UMPS catalyzes the last two steps of the *de novo* synthesis of UMP and is central for metabolizing the chemotherapeutic drug 5-FU into its active metabolites. As a result, cells lacking BOK have a defect in uridine metabolism, leading to decreased cellular proliferation compared to WT controls. On the other hand, *Bok^– /–^* cells are resistant toward 5-FU treatment. Losing BOK seems to confer a selective advantage to CRC cells, and consequently, BOK expression levels are suggested to be useful as a prognostic marker in CRC and as a predictive marker for the efficacy of 5-FU treatment in CRC. While there is increasing evidence for a UPR-modulating role for BOK, the exact mechanism and possible interaction partners for UPR modulation and the ER stress response are still unknown.

So far, only a few BOK interacting partners have been described (Figure 1). Using yeast two-hybrid screens, BOK was found to interact with some selected antiapoptotic members of the BCL-2 family, i.e., MCL-1, BFL-1/BCL-2A1, and the Epstein-Barr virus BCL-2 homolog BHRF1, but neither with BCL-2, BCL-XL, or BCL-W, nor with BAX or BAK (Hsu et al., 1997; Srivastava et al., 2019). Using co-immunoprecipitation assays of overexpressed proteins, Echeverry et al. reported failure of BOK to interact with any tested BCL-2 family member (BCL-2, BCL-XL, MCL-1, BAX, BAK), with the exception of BOK itself, an interaction that is dependent on critical amino acids within the BH3 domain (Echeverry et al., 2013). However, there currently are no convincing data published that BOK forms dimers or higher homo-oligomers or hetero-oligomers. Moreover, both yeast two-hybrid screens and co-immunoprecipitation assays are error-prone; thus, more reliable assays are needed to clarify the interactions of BOK with other family members. Like most BCL-2 proteins, BOK contains a C-terminal TMD that serves as a tail-anchor domain for the posttranslational insertion at the cytoplasmic side of intracellular membranes (Hsu et al., 1997; Schinzel et al., 2004). Tail anchors are both necessary and sufficient to target a protein to the ER, the MOM, or to other intracellular membranes, whereas they are not always specific for a single compartment with known examples of dual targeting of ER and MOM (Borgese and Fasana, 2011). Echeverry et al. (2013) have demonstrated that the TMD of BOK has a high affinity for the ER, Golgi, and associated membranes, into which it integrally inserts. However, whereas the majority of BOK is located at the ER, BOK can also be found at the mitochondria (Ray et al., 2010; Echeverry et al., 2013; Einsele-Scholz et al., 2016; Ausman et al., 2018) and has further been reported to localize to the nucleus, where its role is still rather enigmatic (Bartholomeusz et al., 2006; Echeverry et al., 2013). In contrast to most other multi–BH-domain BCL-2 proteins, which contain a classic hydrophobic TMD, the TMD of BOK is peculiar in that it comprises two positively charged amino acids (aa) in its center (R^200^ and K^203^ in mouse BOK) (Lindsay et al., 2011). The functions of these polar aa are not clear, but may affect the topology of the BOK TMD and its membrane insertion; on the other hand, it may also be speculated that these polar aa mediate intramembranous homodimerization or heterodimerization with polar TMDs of interaction partners.

**FIGURE 2:**
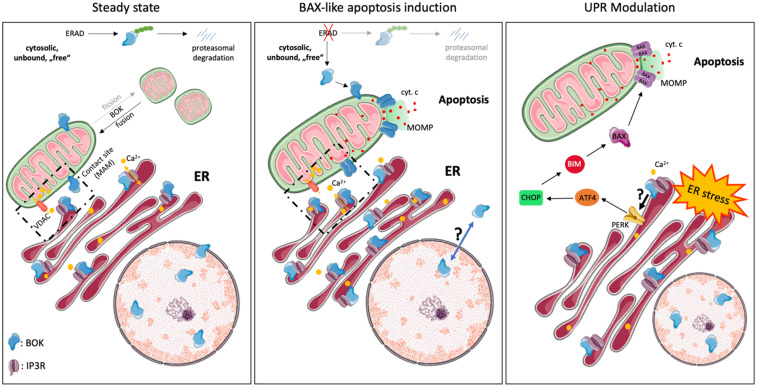
Intracellular localization and cellular functions of BOK. BOK was described to exert various cellular functions at steady state and upon stress exposure, probably dependent on its various subcellular localizations. At steady state, the majority of BOK is bound to the ER, Golgi, and associated membranes. However, it can also be found within the nucleus, where its role is unclear, as well as on the MOM and ER-mitochondrial contact sites. ER-localized BOK is bound to IP3R and stabilizes the latter from caspase-3–mediated cleavage. IP3R-bound BOK, on the other hand, is stabilized from proteasomal turnover, as unbound, “free” BOK is ubiquitinated and degraded by the ERAD pathway. IP3R-bound BOK may thus not be available for mitochondrial apoptosis induction. No direct effect of BOK in altering Ca^2+^ mobilization by IP3Rs has been observed so far. Recently, BOK was found to be important for mitochondrial fission and morphology and thus to affect mitochondrial membrane potential. BOK has furthermore been described to exert a BAX-like killing function. Disturbance of components of the ERAD pathway thereby results in the stabilization of “free” BOK protein, which then directly induces MOMP and apoptosis in a BAX-like manner, independently of other BCL-2 family members. Under certain stress conditions, e.g., under hypoxia, BOK is upregulated and translocates from the nucleus into the cytoplasm and to mitochondrial membranes by a still unknown mechanism, where it may induce apoptosis. Increasing evidence points toward a UPR-modulating function by BOK, following ER stress, by a yet unknown mechanism. In that respect, BOK was described to regulate the PERK > ATF4 arm along with CHOP, which induces the BH3-only protein BIM (and PUMA). BIM directly activates BAX/BAK, leading to MOMP and subsequent apoptosis, suggesting an apoptosis-inducing role of BOK upstream of MOMP in that context.

BOK’s dominant localization at the ER is an important distinction from BAX or BAK and is much in line with increasing evidence for a role of BOK in the modulation of ER stress responses (see below). This is also strongly supported by the direct interaction of BOK with inositol 1,4,5-trisphosphate receptors (IP3R), in particular with IP3R1 and IP3R2, as identified by the Wojcikiewicz laboratory (Schulman et al., 2013, 2016). This interaction was shown to be strong and constitutive and mediated by the N-terminal BH4 domain of BOK binding to a region within the IP3R coupling domain, a region to which no other BCL-2 family member has been reported to bind. Through this interaction, BOK protects IP3R from caspase-3–mediated degradation during apoptosis (Schulman et al., 2013). However, no direct effect of BOK in altering Ca^2+^ mobilization by IP3Rs was observed (Schulman et al., 2013). Nevertheless, a role for BOK in maintaining Ca^2+^ homeostasis in response to excitotoxic stimulation has been described in neurons, and this phenotype was linked to a decreased protein stability of MCL-1 observed in the absence of BOK (D’Orsi et al., 2016). Importantly, Schulman et al. (2016) showed that BOK is stabilized when bound to IP3R and that its proapoptotic activity is thereby likely limited, whereas unbound (“free”) BOK is rapidly degraded by the ubiquitin proteasome pathway. In consideration of the potent proapoptotic activity of BOK reported by Llambi et al. (2016), it can be argued that IP3Rs may act as a regulatory sink for BOK to keep the levels of unbound (“free”) BOK low (Figure 2). Furthermore, Schulman et al. (2016) also identified an alternative start site for BOK at Met^15^, resulting in a shorter protein that should not be mistaken for BOK’s shorter isoform, BOK-S. Regulation and functional relevance of the resulting N-terminally truncated BOK protein is currently unknown and needs further investigation.

Recently, BOK was identified to interact with uridine monophosphate synthetase (UMPS) (Figure 1; Srivastava et al., 2019). UMPS, which in higher eukaryotes is a bifunctional enzyme consisting of the orotate phosphoribosyltransferase (OPRTase) and orotidine decarboxylase (ODCase) domains, is a central enzyme catalyzing the final steps in the *de novo* synthesis of orotate to uridine monophosphate (UMP) (Qumsiyeh et al., 1989). BOK binds to the ODCase domain at its dimer interface in a BH3-dependent manner (Srivastava et al., 2019). Intriguingly, it was found that, through this interaction, BOK increases the enzymatic activity of UMPS approximately threefold (Srivastava et al., 2019). As a consequence, cells lacking BOK have a defect in uridine metabolism, which affects crucial metabolic pathways including pyrimidine nucleotide synthesis, resulting in decreased cellular proliferation of *Bok^–/–^* cells compared with WT controls (Rabachini et al., 2018; Srivastava et al., 2019). On the other hand, loss of BOK confers resistance to 5-fluorouracil (5-FU)–induced apoptosis, as UMPS catalyzes the biotransformation of 5-FU into its active downstream metabolites. As discussed below, there are important implications of these findings for cancer biology, as colorectal cancer (CRC) cells may have a selective advantage from losing BOK expression (Srivastava et al., 2019). The study by Srivastava et al. provides the first link between BOK and uridine metabolism. Along that line, a connection from BOK to mitochondrial bioenergetics, fusion/fission and morphology has recently been reported also, further supporting apoptosis-independent roles of BOK (D’Orsi et al., 2016; Ausman et al., 2018; Schulman et al., 2019). Taken together, there is solid evidence published for the interaction of BOK with IP3R1/-2, as well as with UMPS. Regarding the BCL-2 proteins, the best evidence for interaction is with MCL-1 (Figure 1).

## Various Models of BOK-Induced Apoptosis

**FIGURE 3:**
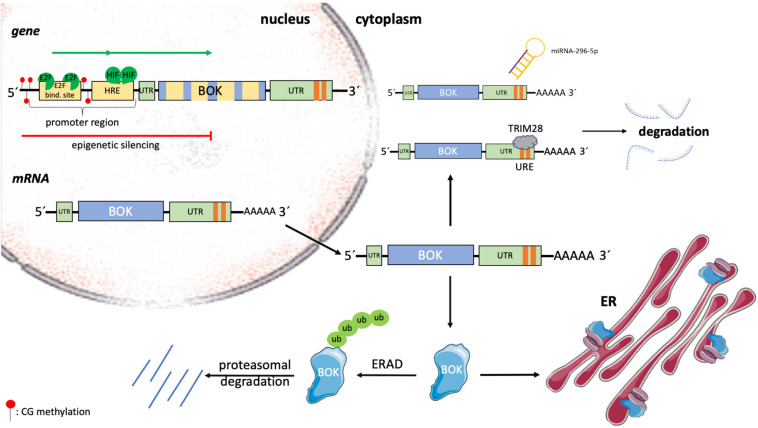
Transcriptional and posttranslational regulation of BOK. The expression of BOK is regulated at transcriptional, epigenetic, translational, and posttranslational levels. In a variety of human cancers, the genomic region containing the BOK locus on chromosome 2 is deleted. Within its promoter region, BOK contains HREs, as well as a conserved E2F1-binding site. Upon binding by hypoxia-inducible factors (HIFs) and the cell cycle–regulating transcription factor E2F1, respectively, BOK transcription is induced. On the other hand, by means of promoter methylation, BOK can be epigenetically silenced. BOK comprises five exons. Alternative splicing leads to the development of the poorly understood BOK isoforms BOK-S and BOK-P. The expression of BOK is furthermore regulated at the level of mRNA stability. The miRNA miR-296-5p has been shown to bind to the 3’ UTR of BOK mRNA, leading to its degradation. Moreover, strongly destabilizing (AU/U)–rich elements (one ARE site in mouse *Bok* mRNA, two URE sites in human *BOK* mRNA) were described in the 3’ UTR of BOK. Tripartite motif containing 28 (TRIM28) was identified to bind to this region, leading to destabilization and subsequent degradation of human *BOK* mRNA. Once translated, BOK is mainly localized at ER membranes, where it is bound to IP3Rs. Unbound BOK is quickly turned over by the ERAD pathway, resulting in ubiquitin-proteasomal degradation.

The role and exact molecular function of BOK in apoptosis is still not fully understood. Since its discovery, BOK has been grouped together with BAX and BAK as “killer”; however, whether BOK induces MOMP in the absence of BAX or BAK and how relevant BOK-induced apoptosis is for (patho)physiology are currently controversially discussed. Earlier reports provided evidence that BOK induces intrinsic apoptosis, but these studies were restricted to transient overexpression and were performed in BAX/BAK-proficient cell lines (Hsu et al., 1997; Yakovlev et al., 2004; Rodriguez et al., 2006). Whereas it seems to be clear that high levels of BOK can induce apoptosis, two studies reported that cell death induced by overexpressed BOK is much reduced in *Bax^–/–^Bak^–/–^* DKO cells (SV40-immortalized MEF, Hoxb8-immortalized growth factor–dependent myeloid progenitors) compared to WT controls (Echeverry et al., 2013; Carpio et al., 2015). These data implied that BOK may act upstream of BAX/BAK-mediated MOMP, which is in line with its predominant subcellular localization at the ER and its role in modulating ER stress responses (Echeverry et al., 2013; Carpio et al., 2015; Llambi et al., 2016). On the other hand, several studies reported that increased BOK levels are able to kill *Bax^–/–^Bak^–/–^* DKO cells (HCT116, SV40 MEF) and to induce MOMP in the absence of BAX and BAK, implying a true BAX-like effector function of BOK at the MOM (Figure 2; Einsele-Scholz et al., 2016; Llambi et al., 2016; Zheng et al., 2018). While these latter reports are in strong support for BOK-induced MOMP, biophysical evidence for an oligomerization of BOK and direct pore formation in the MOM is currently missing. Of note, while recombinant forms of BOK readily permeabilized artificial membrane vesicles, in particular when their composition mimicked the MOM, it failed to do so on isolated mitochondria lacking BAX and BAK, even in the presence of the activator BH3-only protein tBID (Fernandez-Marrero et al., 2017). A possible alternative explanation was put forward by Llambi et al. (2016) and Zheng et al. (2018), who proposed a unique mechanism for BOK-induced MOMP, which is independent of interaction with tBID or other BCL-2 family members. Interestingly, this mechanism relies on an intrinsic instability of BOK (hydrophobic groove and helix α1), which results in its spontaneous association with mitochondria and MOMP, independent of the activation by BH3 ligands of other BCL-2 family members. Because of this model, BOK is expected to be a very potent inducer of MOMP and apoptosis, and thus, BOK levels need to be tightly controlled. One way to control BOK levels is through ER-associated degradation (ERAD), with a high turnover of BOK via ubiquitination and proteasomal degradation (Figures 2, 3; Llambi et al., 2016). However, several reports have shown that many cell types or tissues can tolerate high levels of BOK protein (Ke et al., 2012; Moravcikova et al., 2017; Srivastava et al., 2019; Zhang et al., 2019), indicating that other posttranslational mechanisms are in place to control BOK activity and/or to sequester it away from mitochondria.

Whether loss of BOK protects cells from specific apoptotic stresses cannot be generalized and may be highly specific on the cell type or the nature of the apoptotic stress. Ke et al. (2012) showed that hematopoietic cells derived from *Bok^–/–^* mice are normally sensitive to dexamethasone, etoposide, FasL, or ABT-737–induced apoptosis. Likewise, various cell types, including MEF, were shown to respond normally to etoposide or staurosporine in the absence of BOK (Echeverry et al., 2013; Carpio et al., 2015; Fernandez-Marrero et al., 2016). On the other hand, however, Einsele-Scholz et al. (2016) reported that knocking down BOK leads to decreased drug-induced (taxol, cisplatin, camptothecin) cell death of ovarian carcinoma cells, OVCAR-3, OVCAR-4, and OVCAR-8. This seeming discrepancy in the involvement of BOK in response to DNA damaging agents may be explained by cell-intrinsic differences (e.g., amount of endogenous BOK, cancer cells vs. normal cells) and requires further investigation. However, what seems to be emerging is that BOK has important functions at the ER in response to ER stress (Echeverry et al., 2013; Carpio et al., 2015; Moravcikova et al., 2017). Carpio et al. (2015) described BOK as a promoter of cell death in SV40-immortalized MEF in response to various kinds of ER stress. The authors reported that BOK connects ER stress signaling to the intrinsic apoptotic machinery through modulating the UPR, in particular the PERK > ATF4 arm along with CHOP, which induces the BH3-only protein BIM (Carpio et al., 2015). Similarly, Kang et al. (2019) found decreased ATF4 and CHOP expression in erythrocytes derived from *Bok^–/–^* mice compared to WT mice, also suggesting a role for BOK in modulating the UPR in response to ER stress. In line with these findings, Rabachini et al. (2018) showed that chemical [diethylnitrosamine (DEN)]–induced upregulation of CHOP and BIM, and consequently hepatocellular apoptosis, is reduced in livers of *Bok^–/–^* mice. These studies are supportive of a proapoptotic role of BOK at the level of the ER and upstream of mitochondrial apoptotic events (Figure 2). It should also be mentioned that, in some instances, BOK has been attributed a protective or apoptosis-unrelated role in response to ER stress or other stressors and has also been shown to affect other arms of the UPR, namely, the IRE1α branch (Echeverry et al., 2013; D’Orsi et al., 2016). Taken together, based on the current literature, there is increasing evidence to put an important place of action of BOK at the ER membrane and it seems that—at the ER—BOK may be best described as a modulator of UPR signaling in response to ER stress (Carpio et al., 2016; Fernandez-Marrero et al., 2016). Whereas most of the previously described effects were derived from cell lines, the recent study by Ke et al. (2018) provides the up to now most convincing evidence for a relevant proapoptotic role of BOK *in vivo*. One key finding of that study was a more severe developmental phenotype in *Bax^–/–^Bak^–/–^Bok^–/–^* full-body TKO mice compared with *Bax^–/–^Bak^–/–^* DKO controls, demonstrating overlapping roles of BOK with BAX and BAK during mouse embryogenesis.

## Regulation of BOK Expression

Regulation of BOK expression in health and disease has been described at transcriptional, epigenetic, and posttranslational levels, but is overall only incompletely understood (Figure 3). Within the promoter of human and mouse BOK, a conserved E2F-binding site has been described (Rodriguez et al., 2006; Luo et al., 2014). Consequently, Rodriguez et al. (2006) described BOK to be cell cycle regulated by the transcription factor E2F1, which upon binding to the promoter activates transcription. E2F1 plays a crucial role in the control of the cell cycle but is not only involved in cellular proliferation but also regulates the expression of BH3-only proteins such as BIM and NOXA and furthermore mediates p53-dependent apoptosis (Hershko and Ginsberg, 2004; Zhang et al., 2020). In the model of Rodriguez et al., starvation followed by serum treatment, which stimulates quiescent cells to enter S phase, and overexpression of E2F1, respectively, led to an increase of *BOK* mRNA in H1299 cells (Rodriguez et al., 2006). In contrast to the above findings, Ha et al. (2001) found an increase in *BOK* mRNA upon serum starvation, rather than serum stimulation in HC11 mammary epithelial cells as well as in mouse mammary glands. Yet, the mechanism behind the observed BOK upregulation in this report has not been resolved. In all cases, however, the increase in BOK was correlated with an increase in apoptosis. But, while apoptosis induction in the mouse mammary gland was described as a physiological process after weaning, the increased induction of apoptosis in H1299 cells was observed only in response to an extrinsic stressor (Ha et al., 2001; Rodriguez et al., 2006). Within the *BOK* promoter region, Luo et al. (2014) described the presence of hypoxia response elements (HREs). Hypoxia-inducible factors play an important role in adapting the cellular metabolism in response to cellular stress caused by hypoxia (Vanderhaeghen et al., 2020) and were shown to bind to HRE in the promoter region of BOK, leading to an induction of BOK expression and increase in mitochondrial apoptosis (Soleymanlou et al., 2005, 2007; Luo et al., 2014). Furthermore, the expression of BOK, as well as of BAX, has been reported to be controlled by Wnt signaling in intestinal cancer (Zeilstra et al., 2011). Dysregulated Wnt signaling, which is frequently observed in sporadic CRC patients, was correlated with an increase in *BOK* and *BAX* mRNAs and accordingly with increased apoptotic activity (Zeilstra et al., 2011).

In a variety of human cancers, BOK is frequently found to be downregulated. Often, this downregulation can be associated with the deletion of the genomic region containing the BOK locus (Beroukhim et al., 2010). Moreover, epigenetic silencing by promoter methylation has been described in non–small cell lung carcinoma (NSCLC) (Moravcikova et al., 2017). In colorectal carcinoma tissue, in which BOK protein was also found to be decreased compared to matched healthy tissue, promoter hypomethylation rather than hypermethylation has been observed, thus excluding epigenetic downregulation in CRC (Carberry et al., 2018; Srivastava et al., 2019). Hence, so far, only little is known about the epigenetic regulation of the *BOK* gene.

At the level of mRNA stability and translation, *BOK* was found to be downregulated by binding of the miRNA miR-296-5p to its 3’ UTR in breast cancer cells (Onyeagucha et al., 2017). Furthermore, destabilizing (AU/U)–rich elements [one AU-rich element (ARE) site in mouse *Bok*, two U-rich element (URE) sites in human *BOK*] were recently described in the 3’ UTR of BOK (Fernandez-Marrero et al., 2018). In that study, the authors identified tripartite motif containing 28 (TRIM28), a large pleiotropic protein that is known for its many roles at the genomic level in the nucleus (Czerwinska et al., 2017), as a novel URE-binding protein triggering the degradation of human *BOK* mRNA (Figure 3; Fernandez-Marrero et al., 2018).

At the protein level, little is known about BOK activation or how possible changes in subcellular localization are regulated. BOK protein stability is regulated by ubiquitination and proteasomal degradation and may be cell type specific (Schulman et al., 2013, 2016; Llambi et al., 2016; Moravcikova et al., 2017; Sopha et al., 2017). Schulman et al. (2013, 2016) showed that major portions of cellular BOK are constitutively bound to IP3R and protected from degradation, whereas newly synthesized BOK is quickly degraded by the ubiquitin–proteasome pathway. More specifically, BOK is rapidly turned over by the ERAD components AMFR/gp78, by which BOK is ubiquitylated, and VCP/p97, a complex that processes ubiquitylated BOK further for proteasomal degradation. Upon inhibition of ERAD, or of proteasome activity, BOK protein gets stabilized and induces MOMP (Llambi et al., 2016). Whether and how BOK translocates from the ER to mitochondria are not known. One possibility is that BOK is already present at mitochondria–ER contact sites, such as mitochondria-associated membranes (MAM), which again needs further investigation (Figure 2; Giacomello and Pellegrini, 2016).

## Role of BOK in Pathophysiology and Cancer

There is increasing evidence pointing toward BOK as a prognostic or predictive marker in cancer. Interestingly, the genomic region containing BOK was identified to be relatively frequently deleted across many types of human cancers (Beroukhim et al., 2010). Given the evidence for a proapoptotic role of BOK, this finding could hint toward a tumor suppressor–like activity of BOK. However, the peak region of the genomic deletion in question contains 18 other genes (Beroukhim et al., 2010) and hard evidence that BOK is indeed a tumor suppressor is currently missing. Nevertheless, the finding that BOK is one of the most frequently deleted genes among the proapoptotic family members was unexpected and merits further investigation. Besides loss of BOK through somatic copy-number variations, epigenetic repression of BOK expression was proposed from studies in NSCLC cell lines (Moravcikova et al., 2017). Here, analysis of primary NSCLC patient samples revealed that BOK protein levels are reduced in NSCLC tumors compared with matched healthy lung tissue. Importantly, in poorly differentiated tumors, as well as in tumors of patients with lymph node infiltrations and metastases, BOK protein was even further decreased. For those patients, a weak positive correlation between BOK protein and overall survival was identified. Hence, these data do support a tumor-suppressing action of BOK and suggest that BOK protein levels may be useful as a prognostic marker in late-stage NCSLC patients (Moravcikova et al., 2017). Of note, that study also provided evidence that BOK does not directly induce apoptosis or affect apoptotic responses toward chemotherapeutic drugs, but rather that high BOK levels antagonize transforming growth factor β–induced epithelial-to-mesenchymal transition and may thus slow down malignant transformation (metastasization) of NSCLC. Mechanistically, it was suggested that this function is mediated via the modulation of the PERK > ATF4 arm of the UPR, a function of BOK first described by Carpio and colleagues (Carpio et al., 2015; Moravcikova et al., 2017). In line with this, Kang et al. (2019) described BOK to be important for erythropoiesis in the context of Nup98-HoxD13 (NHD13)–driven myelodysplastic syndrome. In this study, the induction of ATF4 and CHOP was significantly reduced in erythrocytes derived from *Bok^–/–^* mice compared to WT mice. While erythropoiesis in *Bok^–/–^* mice was not affected compared to WT mice, NHD13 transgenic mice showed reduced BFU-E (blast-forming units–erythroid) colonies and a progressive anemia when BOK was lost (Kang et al., 2019). It was concluded that BOK contributes to erythropoiesis under stress conditions, probably via modulation of the ATF4 pathway (Table 1; Kang et al., 2019).

BOK mRNA stability is negatively regulated by the presence of one ARE in the mouse and two URE in the human 3’ UTR (Fernandez-Marrero et al., 2018). TRIM28 was identified as one of the factors binding to the UREs in human *BOK* mRNA, leading to its degradation. TRIM28 is overexpressed in various cancers in which it is associated with poor prognosis (Czerwinska et al., 2017). Of note, TRIM28 and BOK levels were found to be negatively correlated in selected cancers such as hepatocellular carcinoma and kidney cancer. In those cancers, high *BOK* and low *TRIM28* mRNA levels correlated with increased survival, and low *BOK* and high *TRIM28* levels with decreased survival (Fernandez-Marrero et al., 2018). Translational silencing of *BOK* mRNA by miRNA miR-296-5p was reported by Onyeagucha et al. (2017) in human breast cancer cell lines. This study also describes an miR-296-5p–mediated regulatory feedback loop between *MCL-1* and *BOK* levels that determines whether the breast cancer cells die by apoptosis or survive. Like in NSCLC, BOK protein levels were again found to be significantly lower in tumor tissue compared to matched normal tissue, and higher BOK expression positively correlated with overall as well as with relapse-free survival of breast cancer patients (Onyeagucha et al., 2017).

Besides NSCLC and breast cancer, BOK has been shown to be downregulated in CRC and to be of potential prognostic and predictive value, respectively (Carberry et al., 2018; Srivastava et al., 2019). Srivastava et al. (2019) reported increased resistance of BOK-deficient CRC cells and MEFs toward 5-FU and showed that CRC cells lose BOK expression when becoming resistant to 5-FU. Mechanistically, that study showed that BOK directly interacts and thereby activates the enzymatic activity of UMPS and provided the first connection of BOK to uridine and nucleotide metabolism (Srivastava et al., 2019). UMPS is a central enzyme in both the conversion of 5-FU into its active metabolites, as well as in the *de novo* synthesis of UMP and hence pyrimidine nucleotides. A consequence of the latter was that BOK-deficient cells show a proliferation defect that is likely caused by an increase in p53 activity at steady state. Importantly, this growth defect of *BOK^–/–^* cells could be rescued by reintroduction of WT BOK, but not of a BOK mutant that fails to interact with UMPS (Srivastava et al., 2019). Overall, this study identified BOK as a double-edged sword in CRC. On the one hand, 5-FU–treated CRC cells acquire a selective advantage by losing BOK; on the other hand, this advantage costs them proliferative potential. The latter may put selective pressure on the BOK-deficient cells to lose cell cycle inhibitors and/or p53 activity, eventually resulting in a faster-growing and likely more aggressive phenotype. BOK is therefore suggested to serve as a predictive marker in CRC, and the authors proposed to explore the potential of BOK-mimetic compounds to sensitize resistant CRC to 5-FU treatment (Srivastava et al., 2019). The observation by Carberry et al. (2018) that high BOK levels in CRC surprisingly correlate with reduced overall survival may be dependent on the time of biopsy (tumor stage) and could possibly be explained by the decreased proliferation of BOK-deficient, yet still p53 proficient, tumors.

The role of BOK in affecting cellular proliferation, cancer development, and progression was also reported in a chemical-induced hepatocellular carcinoma model in the mouse (Rabachini et al., 2018). The carcinogen DEN triggers acute hepatic injury and a subsequent inflammatory response that is followed by compensatory hepatocyte proliferation. Regarding the acute phase, livers from *Bok^–/–^* mice were shown to be protected from hepatocellular apoptosis induced by DEN through induction of CHOP, BIM, and PUMA (Rabachini et al., 2018). These data provided evidence for a proapoptotic role of BOK in a pathophysiologically relevant setting, whereas it suggested at the same time an “indirect” killing mechanism, with BOK inducing cell death upstream of CHOP and BH3-only proteins, and thus likely upstream of MOMP. Because of the protection during the acute phase, *Bok^–/–^* mice developed fewer tumors at the endpoint of 9 months. Of note, the tumors in *Bok^–/–^* mice were also smaller and showed a reduced proliferative index. Furthermore, BOK-deficient HCC cells and MEF proliferated slower compared to BOK proficient cells, which is in line with the growth defect reported in the study of Srivastava et al. (2019) and others (Ray et al., 2010; Moravcikova et al., 2017; Zhang et al., 2019).

Besides cancer, BOK expression and regulation have been connected with preeclampsia, a placental disease that may occur during pregnancies and that is characterized by a reduced oxygenation of trophoblast cells, increased trophoblast cell death, and trophoblast hyperproliferation (Chaiworapongsa et al., 2014). The Caniggia laboratory reported physiological BOK expression in proliferating trophoblast cells that is further increased in preeclampsia, where it contributes to the hyperproliferative state of the trophoblasts (Ray et al., 2010). Of note, in this context, it seems that nuclear localized BOK is important for cellular proliferation of trophoblast cells during placental development, while cytoplasmic BOK induces cell death (Ray et al., 2010). Moreover, in patients suffering from preeclampsia, a splice variant of BOK, BOK-P, was described (Soleymanlou et al., 2005; Ray et al., 2010). BOK-P results from skipping exon 2 (Soleymanlou et al., 2005), which contains the first of the two existing ATG start codons within the human *BOK* gene (a second start codon is localized within human, but not mouse, exon 3). The resulting BOK isoform lacks the BH4 and parts of the BH3 domain, as well as parts of the 5’ UTR (Soleymanlou et al., 2005). Apart from cellular proliferation (Ray et al., 2010) and apoptosis induction (Soleymanlou et al., 2005), several observations point toward an autophagy regulating role of BOK in preeclampsia (Kalkat et al., 2013; Melland-Smith et al., 2015; Ausman et al., 2018). In this setting, upregulation of BOK due to hypoxic conditions (Kalkat et al., 2013) or due to ceramide accumulation within the mitochondria (Melland-Smith et al., 2015; Ausman et al., 2018) leads to an increase in autophagic flux (Kalkat et al., 2013; Melland-Smith et al., 2015; Ausman et al., 2018). The involvement of BOK in autophagy regulation can be explained by the observation that BOK disrupts the interaction between MCL-1 and Beclin-1, which ultimately leads to the induction of autophagy (Kalkat et al., 2013). Beclin-1 has been described to interact with different antiapoptotic members of the BCL-2 family of proteins via its BH3 domain, namely, with MCL-1, BCL-2, and BCL-XL (Marino et al., 2014). These interactions can be disrupted by several BH3-only proteins of the Bcl-2 family–like NOXA and PUMA (Marino et al., 2014) and supposedly also by BOK (Kalkat et al., 2013), leading to an increase in freely available Beclin-1 and subsequent autophagy induction (Kalkat et al., 2013; Marino et al., 2014). However, the model that the interaction of different BCL-2 family members with Beclin-1 is sufficient to regulate autophagy under physiological conditions has been challenged (Lindqvist et al., 2014). Lindqvist et al. (2014) proposed that endogenous levels of the BCL-2 family proteins are not able to directly influence autophagy, since the binding of Beclin-1 with the BH3 domain of different BCL-2 family members is rather weak and thus insufficient to lead to the sequestration of Beclin-1. They concluded that the impact of the BCL-2 family on autophagy is indirect through BAX and BAK inhibition by prosurvival BCL-2 family members rather than direct through effects on Beclin-1 (Lindqvist et al., 2014). In their model, the inhibition of BAX/BAK and the resulting inhibition of apoptosis lead to the activation of autophagy by a yet unknown mechanism (Lindqvist et al., 2014). Nevertheless, given that several other groups ascribe a role of various members of the BCL-2 family not only to apoptosis but also to autophagy induction and repression, respectively, it seems likely that BOK may likewise play a role in the regulation of autophagy initiation. Whether this regulation is important under physiological or only under pathophysiological conditions needs further investigation.

## Conclusion

Over the last years, various functions have been described for BOK. However, its main function (or functions) remains elusive, and there are still many riddles to be answered. What exactly is its role in apoptosis? Does the function of BOK depend on the intracellular organelle it localizes to, i.e., does it exert different functions at the ER membrane, mitochondrion, or in the nucleus, respectively? What is its role in cancer progression, and why do many types of human cancer lose BOK expression? Does it act as tumor suppressor and, if so, in which cancer subtypes and by what mechanism? Currently, a lot of discordance on BOK and its function can be found in the literature, which makes it difficult to fully grasp the key roles of this protein. In part, this problem may be explained by different cellular systems, mouse models, and different disease settings that have been used by different groups. It, however, also indicates that—more than 20 years after its discovery—there remain lots of open questions and work to be done to understand this fascinating and multifaceted member of the BCL-2 family.

## Author Contributions

TK and SN wrote the manuscript.

## Conflict of Interest

The authors declare that the research was conducted in the absence of any commercial or financial relationships that could be construed as a potential conflict of interest.
